# Robot-assisted simple prostatectomy: the evolution of a surgical technique

**DOI:** 10.1590/S1677-5538.IBJU.2020.0744

**Published:** 2020-12-20

**Authors:** Gilberto J. Rodrigues, Guilherme V. Sawczyn, Giuliano B. Guglielmetti, Arnaldo J. C. Fazoli, Luís H. R. Tanure, William C. Nahas, Rafael F. Coelho

**Affiliations:** 1 Instituto do Câncer do Estado de São Paulo São PauloSP Brasil Instituto do Câncer do Estado de São Paulo, São Paulo, SP, Brasil.; 2 Américas Centro Integrado de Oncologia São PauloSP Brasil Américas Centro Integrado de Oncologia, São Paulo, SP, Brasil.; 3 Hospital Paulistano São PauloSP Brasil Hospital Paulistano, São Paulo, SP, Brasil.; 4 Hospital 9 de Julho São PauloSP Brasil Hospital 9 de Julho, São Paulo, SP, Brasil.

## Abstract

**Purpose:**

Enucleation of a large prostate is the best surgical choice for patients refractory to clinical treatment (1,2). Since the first robot-assisted simple prostatectomy (RASP) was described (3,4), some technical modifications (5–7) and different approaches to reach the adenoma have been proposed (8,9). The aim of this video is to demonstrate three different techniques of RASP.

**Materials and Methods:**

The first procedure begins with a transversal incision over the bladder neck, the second is a transvesical approach and the last one is a Retzius-sparing RASP. All techniques were performed with a vesico-urethral anastomosis.

**Results:**

Three patients underwent RASP, each one with a different approach. Patients presented mean age of 66±4.4 years, PSA baseline level of 7.8±3ng/mL, IPSS score of 17.7±4.5, maximum urine flow of 8.3±1.5mL/seg and 122.3±11.2cm3 of prostate volume. The mean operative time was 63±8 minutes, estimated blood loss of 106.7±11.5mL, prostate weight of the surgical specimen of 106.3±8 grams and 1 day of length of stay. No continuous bladder irrigation was required and there was no complication. The mean postoperative PSA and IPSS were 0.7±0.3ng/mL, 4.7±1.5. The maximum urine flow raised to 20±4.4mL/seg.

**Conclusions:**

RASP with vesico-urethral anastomosis allowed minimal blood loss, short length of stay and great functional outcomes. All the three approaches allowed to perform this technique in a safe way, while showing different alternatives to reach the adenoma.
